# A patient survey of out-of-hours care provided by Emergency Care Practitioners

**DOI:** 10.1186/1471-227X-7-4

**Published:** 2007-06-15

**Authors:** Mary Halter, Tom Marlow, Daryl Mohammed, George TH Ellison

**Affiliations:** 1 Faculty of Health and Social Care Sciences, Kingston University and St George's, University of London, Grosvenor Wing, St George's Hospital, Cranmer Terrace, London SW17 0RE, UK; 2 Community Services Development, 95 Beaconsfield Road, Surbiton, KG5 9AW, UK; 3 London Ambulance Service NHS Trust, 8-20 Pocock Street, London, SE1 0BW, UK

## Abstract

**Background:**

Emergency Care Practitioners (ECPs) have recently been deployed to provide out-of-hours primary care home visits – a practice development that has been supported by policy makers. The aim of the study was to evaluate the care provided to patients receiving out-of-hours home visits from ECPs in London from the patients' perspective and to assess their wellbeing following the visit.

**Methods:**

A bespoke telephone-administered questionnaire was designed to survey all patients who had received out-of-hours care in Bromley Primary Care Trust from ECPs during a ten week period in 2005 (n = 174).

**Results:**

Sixty three patients (36.2%) were excluded because: no telephone number was available; they had a diagnosis of dementia; or had not received a study information sheet. The remainder (n = 111) were contacted 3–5 days after the home visit, and 81 of these (73.0%) completed the survey. Of those respondents treated at home who gave unequivocal answers (n = 60), all but one (8.3%) reported that they felt that their treatment had been 'right' and/or had followed any advice given. However, overall only 86.4% reported that they had been clear about their ECP's assessment, and only 58.0% reported that their health was now 'better'. Those who reported that they were *not *clear about their assessment were less likely to report that their health was 'better' (*p *= 0.03) and more likely to have subsequently used hospital-based health services (*p *= 0.03).

**Conclusion:**

Most patients treated at home by ECPs appeared satisfied and compliant with the care provided, according to the measures used in this study. However, it appears that a sizeable minority of patients were unclear about ECP assessments and it remains to be seen whether these patients had pre-existing health complaints which made them less likely to recover and more likely to seek hospital care, or whether the lack of clarity about their assessment undermined their subsequent recovery and necessitated hospital care. Further research is required to establish if the assessments provided by ECPs are less clear than those provided by other practitioners, and whether it is possible to ensure that all such assessments are clear to all patients.

Patients hold a mainly positive view of out-of-hours home visit care provided by ECPs, although a lack of clarity about their assessment was evident, with a possible impact on their continuing health.

## Background

The delivery of out-of-hours primary health care has changed substantially in recent years and now includes a range of service models, including: deputising services; telephone triage; primary care centres; walk-in centres; emergency departments; and/or cooperatives [1]. A new model, which has been positively received by policy makers [2,3], involves Emergency Care Practitioners (ECPs) working with GPs, particularly in the delivery of primary care through out-of-hours home visits. For this and their other enhanced roles, ECPs undertake additional training to develop autonomous practice which aims to enable them to assess and treat the patient at the point of access (wherever possible) and thereby avoid the use of hospital-based emergency departments (wherever appropriate), with subsequent gains in capacity in the hospital sector. Additionally, in the GP environment, the aim has been to reduce waiting times in primary care by visiting patients for GPs [2].

There has been a paucity of research into alternative models of out-of-hours primary care, leading to a lack of evidence about clinical outcomes [4]. What studies there have been have focused on patient satisfaction and have found that patients were less satisfied when they did not receive the care they were expecting [5-7]. Related research on alternative models of pre-hospital emergency care, where Emergency Medical Technicians or Paramedics have adopted an ECP-type role (which allowed them to 'treat and release' patients at the scene – albeit without the additional training that ECPs receive), suggested that there were a number of unresolved concerns about patient safety [8-10].

The aim of the present study was therefore to evaluate the care provided to patients receiving out-of-hours home visits from ECPs in London from the patients' perspective and assess their wellbeing following the visit. We anticipated that the potential existed for ECPs to make inappropriate care decisions, given that ECPs are comparatively inexperienced providers of out-of-hours care. And although this was not intended to be a survey of patient satisfaction, we expected that patients' experiences of care might be negatively affected by the fact that this was delivered by an ECP, given that patients who call their out-of-hours GP service would not have expected an ECP to carry out their home visit.

## Methods

### Study setting

The ECPs currently practising in London are all Emergency Medical Technicians or Paramedics who have undertaken additional diploma-level training, part-time over a two year period. During this time they continue to practice, primarily by responding to emergency ambulance calls. The diploma involves dedicated modules on physical assessment, clinical decision-making, minor illness, chronic conditions, pharmacology, paediatric care and the health of older people. At the same time, ECPs also undertake supervised clinical placements with a variety of other practitioners as part of their continuing professional development, including: GPs; emergency department physicians; community nurses; and social care professionals. More recently they have started to use 'Patient Group Directives' to administer a limited number of medicines, although this had not been introduced at the time the present study took place.

Since December 2004 ECPs have also been deployed to conduct out-of-hours home visits between 09h00 and 21h00 at weekends and on public holidays. When the present study took place (February to April 2005) London ECPs were working alongside GPs in just one such out-of-hours service, in a single NHS-defined geographical area – Bromley Primary Care Trust. This service involves patients who contact their out-of-hours GP service by telephone. Each such call is logged by a call handler who passes the call on to a GP based at the out-of-hours primary care centre. This GP telephones the patient back to elicit any clinical information required and to make an initial assessment of any clinical needs. At this point the patient may be given advice on the telephone, or asked to attend the out-of-hours primary care centre. But if a home visit is deemed necessary, the GP decides if the patient's condition is suitable for an ECP, or whether a GP is required (although the patient will not ordinarily know which type of practitioner will be visiting them). This decision is based on strict criteria, developed by the GP lead for the ECP out-of-hours scheme, which determines which conditions are suitable for home visits by ECPs.

At the time of the present study any information provided by the patient calling the out-of-hours service, together with the assessment made by the GP based at the out-of-hours centre, was transmitted electronically to a computer in the ECP's response car. And after every home visit, whether by a GP or ECP, the patient's own GP received a faxed copy of the 'call sheet' which contained the attending practitioner's record of the assessment they had made, any treatment they had provided and any further care they recommended. This could include a recommendation to the patient's GP that they should be followed up urgently, but this was not always the case. The management information available for analysis in the present study therefore included: the age and gender of the patient; their presenting complaint; subsequent disposal (i.e. treatment at home with/without referral, or conveyance to the out-of-hours primary care centre/hospital emergency department); and the time spent with the patient.

### Survey questionnaire

A bespoke questionnaire was developed by members of the research team (MH and TM), drawing on discussions with the ECP-, GP- and management-leads for out-of-hours care in Bromley Primary Care Trust, and focusing on concerns raised before the scheme became operational. The questionnaire was subsequently examined by these leads who concluded that it had both face and content validity, and had successfully covered all of their principal concerns. The questionnaire used both closed- and open-ended questions and focussed on the following aspects of out-of-hours care: what had happened to the patient during and after the ECP's visit, with questions tailored to two groups -those patients who had received advice (had they followed the advice) and those who had been treated, referred or taken to another facility (had this treatment felt 'right'); clarity about the ECP's assessment (i.e. what the outcome of care would be and when any subsequent care might happen); and whether their health had felt 'better' following the ECP's visit. Data collected during the first week of the study were used to pilot and evaluate the questionnaire, but no issues arose with administering the survey during this period, and since no modifications were made, the data from this initial week were included in the final analyses.

### Participant recruitment

All patients who had received an ECP out-of-hours home visit between 26^th ^February and 15^th ^May 2005 were eligible for recruitment into the study. ECPs were asked to give these patients an information sheet at the end of their home visit, which explained that they might receive a telephone call from a researcher, at which point the research governance approval allowed the researchers to contact the participants and request informed consent to participate in the study. The records of each of these patients were then collected from the GP out-of-hours centre and potential participants were telephoned during office hours within 3–5 days of their ECP home visit (i.e. as soon as possible thereafter).

### Analysis

Responses to the questionnaire were transcribed verbatim during each telephone interview, and were subsequently categorised by two of the authors (MH and TM) prior to analysis. Data were managed in Microsoft Access and were statistically analysed using SPSS version 12 to conduct *t *tests, *χ*^2 ^tests and Fisher's exact tests.

### Research governance

Research governance approval for the study was granted by the London Ambulance Service and Bromley Primary Care Trust.

## Results

Of the 174 eligible participants, 63 (36.2%) were excluded because: they had a diagnosis of dementia (n = 2); no telephone number was available (n = 6); or there was no record that they had received a study information sheet (n = 55; see Table 1). There were no significant differences in age (*χ*^2 ^= 2.76, *d.f*. = 1, *p *> 0.05), gender (*χ*^2 ^= 0.02, *d.f*. = 1, *p *> 0.05), disposal (*χ*^2^<0.01, *d.f*. = 1, *p *> 0.05) or visit duration (*t *= 1.07, *n *= 112, *p *> 0.05) between patients included and excluded from the survey. However, there were significant differences in presenting complaint (*χ*^2 ^= 29.20, *d.f*. = 20, *p *= 0.02), with fewer of those excluded having urological and pain-related complaints and fewer of those included having neurological and gastrointestinal complaints.

**Table 1:** Reasons for excluding potential participants and non-respondents

**Exclusion stage**	**Reason for exclusion**	**n**	**% within stage**
Prior to contacting patients (of n = 174)	No telephone number recorded	6	3.5
	Dementia documented	2	1.1
	No record that the patient had received a study information sheet^1^	55	31.6
	
	Subtotal	63	36.2

On patient contact (of n = 111)	No reply to telephone call	29	26.1
	Patient declined to participate	1	0.9
	
	Subtotal	30	27.0

^1^Careful examination of patients who had not received a study information sheet found that the majority had received visits on seven specific days within the thirty day study period, suggesting that these exclusions reflect a general failure to deliver study information sheets rather than the selective exclusion of specific types of patients during the course of the study.

Only one of the 111 patients included in the study declined to take part, but 29 others failed to answer their phone call (see Table 1). Nonetheless, there were no significant differences in age (*χ*^2 ^= 3.85, *d.f*. = 1, *p *< 0.05), gender (*χ*^2 ^= 0.81, *d.f*. = 1, *p *< 0.05), type of complaint (*χ*^2 ^= 25.2, *d.f*. = 18, *p *< 0.05), or visit duration (*t *= 1.07, *n *= 112, *p *> 0.05) between the 81 participants who completed the questionnaire and the 30 who did not, although the former were significantly more likely to have been treated at home (*χ*^2 ^= 9.09, *d.f*. = 1, *p *< 0.01; see Table 2) and may therefore have been easier to contact by phone within 3–5 days.

**Table 2:** Sociodemographic and service-related characteristics of respondents and non-respondents

**Characteristic**		**Respondents**		**Non respondents**		**All**	
		**n**	**%**	**n**	**%**	**n**	**%**
		
		81	73.0	30	27.0	111	100
Age	Median	82 years		78 years		80 years	
	Aged less than 60	31	39.2	17	60.7	48	44.9
	Aged 60 and over	48	60.8	11	39.3	59	55.1
	Not known	2	-	2	-	4	-

Gender	Female	55	67.9	23	76.7	78	70.3
	Male	26	32.1	7	23.3	33	29.7

Presenting complaint	Respiratory infection	22	27.2	4	13.3	26	23.4
	Urological	14	17.3	6	20.0	20	18.0
	Medical condition^1^	10	12.3	10	33.3	20	18.0
	Back pain	10	12.3	1	3.3	11	9.9
	Gastrointestinal	6	7.4	2	6.7	8	7.2
	Pain (other than chest or back)	5	6.2	2	6.7	7	6.3
	Minor injuries	4	5.0	0	0	4	3.6
	Fainted/dizziness	2	2.5	0	0	2	1.8
	Neurological/stroke	2	2.5	0	0	2	1.8
	Other	6	7.4	5	16.7	11	9.9

Disposal	Treated at home^2^	42	51.9	12	40.0	54	48.6
	Treated at home and referred	26	32.1	5	16.7	31	27.9
	Conveyed to the out-of-hours primary care centre	3	3.7	2	6.7	5	4.5
	Conveyed to the hospital emergency department	10	12.3	11	36.7	21	18.9

Time spent with the patient (minutes)	Median (where known, n = 81)	50 (11–148)		47 (15 – 94)		50 (11–148)	

^1^Medical conditions included arthritis, hypertension, influenza, and heart failure.

^2^Respondents were significantly more likely to have been treated at home (*χ*^2 ^= 9.09, *d.f*. = 1, *p *< 0.01).

From Table 2 it is clear that most of the respondents were female (67.9%) and aged 60 or above (59.3%). More than two thirds had presented with respiratory infections (27.2%), urological complaints (17.3%), back pain (12.3%) or medical conditions (12.3%; including: arthritis; hypertension; influenza; and heart failure), while comparatively few had gastro-intestinal complaints (7.4%), pain (other than chest or back pain; 6.2%), minor injuries (5.0%), dizziness (2.5%) or neurological complaints (2.5%). Few of these conditions resulted in immediate conveyance to the hospital emergency department (12.3%) or out-of-hours primary care centre (3.7%) and most were treated at home, with (32.1%) or without (51.9%) referral to additional services. Finally, there was substantial variation in the duration of ECP home visits, which ranged from just 11 minutes to more than two hours (148 minutes), and averaged just under an hour (50 minutes).

From Table 3, which summarises the experiences of patients captured by the survey questionnaire, it is clear that all but one of those who had not been conveyed to hospital and who had given unequivocal answers (*n *= 59 of *n *= 60; 98.3%) reported that they had felt the treatment they had received had been 'right' and/or had followed the ECP's advice. Nonetheless, within the 3–5 days since their home visit, only 61.5% of the 26 patients that had been referred on by their ECP had actually been seen by these services, and 38.1% of those who had *not *been referred on by their ECP had subsequently been seen by a GP, nurse or hospital-based practitioner. Indeed, overall only 86.4% of the 81 participants reported that they had been clear about their ECP's assessment, and only 58.0% reported that their health was now 'better'. Those who reported that they were *not *clear about their assessment (*n *= 9) were less likely to report that their health was now 'better' (22.2% vs 62.9%; (*χ*^2 ^= 5.41, *d.f*. = 1,  *p *= 0.03), and these respondents were also more likely to have subsequently used hospital-based health services (33% vs 3.3%; *χ*^2 ^= 6.39, *d.f*. = 2, *p *= 0.03).

**Table 3:** Self-reported outcomes following the ECP home visit

**Patient disposal**	**Question**	**Reported outcome**	**n**	**%**
Remained at home (n = 68)	Followed advice given or treatment felt right (n = 68)	Yes	59	86.8
		No	1	1.5
		Unclear from responses	8	11.8
	
	Overall services used (n = 68)	GP or nurse	29	42.6
		Emergency department	1	1.5
		Hospital admission	3	4.4
		None reported	35	51.5
	
	Follow up to the ECP's referral (n = 26)^1^	Not seen yet (GP = 9, physiotherapist = 1)^1^	10	38.5
		Seen and remained at home (GP = 11, nurse = 2, GP and nurse = 1, emergency department = 1)^1^	15	53.8
		Seen and admitted to hospital (GP)	2	7.7
	
	No referral reported but services used (n = 16)	Seen and remained at home (GP = 14, nurse = 1)	15	93.8
		Seen and admitted to hospital (GP)	1	6.2

Conveyed (n = 13)	Outcome following conveyance	Discharged from out-of-hours centre	0	0
		Discharged from the emergency department	3	23.1
		Admitted to hospital	10	76.9

All (n = 81)	Clear about the outcome at the end of the assessment	Yes	70	86.4
		No	9	11.1
		No response to this question	2	2.5
	
	Clear about when the next steps might happen?	Yes	47	58.0
		No	2	2.5
		Unclear from responses	4	4.9
		No response to this question	28	34.6
	
	How are you now?	Better	47	58.0
		Managing	20	24.7
		Needed help	7	8.6
		Worse	7	8.6

^1^One patient was referred to both the hospital emergency department and to a physiotherapist.

## Discussion

Before laying too much store by the results of the present study there are two potential limitations that need to be taken into account. First and foremost, the survey drew on a modest sample of respondents, a disproportionate number of whom were women, treated at home and aged ≥60. However, since older people are the principal recipients of home visits in this out-of-hours service, and since most of those attended at home by GPs remain there after their visit, it seems likely that respondents were broadly similar to patients receiving out-of-hours home visits from GPs. And although there were significant differences in presenting complaint amongst those excluded and included in the study, and it is unclear whether patients selected for ECP visits presented with the same level of complexity or severity of presenting condition, the subsequent diagnoses of respondents in our study suggests that they were broadly representative of patients receiving similar care elsewhere. Indeed, the respondents in the present study had a similar range of diagnoses [11] and outcomes (such as subsequent self-reported health [12] and hospital admission [13]) to those observed by previous studies of out-of-hours care. Nonetheless, the findings of this study are only strictly applicable to patients with similar presenting conditions subject to similar selection for subsequent care, and who were primarily treated at home rather than in hospital.

Meanwhile, a second potential limitation is that the study used a bespoke questionnaire which relied on self-reported outcomes rather than more objective measures which might have provided a more reliable assessment of the quality of care provided by ECPs [14]. However, the use of self-reported outcomes is entirely appropriate for addressing the aim of the present study, which was to assess patients' *experiences *of the care they had received, and this approach was crucial for identifying an important minority of patients who were unclear about the assessments provided by ECPs. As such the questionnaire successfully captured sufficient variation in perceived care and subsequent wellbeing to identify important differences in these outcomes amongst different groups of respondents, even though there was insufficient variation in their response to advice, services used or conveyance to hospital to permit a detailed investigation thereof (see Table 3).

Notwithstanding these potential limitations, the present study does provide a degree of reassurance that ECPs were capable of delivering care that was considered acceptable, or advice that was followed by patients, at out-of-hours home visits, according to the measures we used. Moreover, most of our respondents were treated at home which suggests that our interpretation of findings is particularly relevant to this group. Setting aside the eight respondents who provided equivocal answers, all but one of the remaining 60 respondents who had not been conveyed to hospital felt that the care they had received from their ECP had been 'right' or that they had followed the advice the ECP had given. This high level of agreement with the care provided by ECPs, and of self-reported compliance with the advice offered by ECPs, mirrors the high level of satisfaction found in a recent evaluation of ECPs working in emergency care [15]. This suggests that the ECP model, particularly its focus on treating patients at the point of contact and avoiding conveyance to hospital wherever appropriate, is well-received by most patients in both contexts. And while the present study was not intended to be a survey of general satisfaction with ECP-delivered out-of-hours primary care, it is worth considering such studies in similar settings to inform what we might have expected to find here. In particular, while patient satisfaction is generally higher with home visits than with other models of out-of-hours primary care [4,5], we did not expect such a positive response to the ECP home visit because the patients were likely to have been expecting a GP, and previous studies have found higher satisfaction with out-of-hours care when this met patients' prior expectations [5-7]. With this in mind the present study suggests that patients were sufficiently satisfied with the care received from ECPs to overcome any disappointment at not being visited by a GP.

Despite these reassuring findings, the present study did identify a sizeable minority of respondents who were unclear about their ECP's assessment. These patients were less likely to report that their health was 'better' and more likely to have subsequently sought hospital care. While it is not possible to establish the reasons for these associations from the cross-sectional survey used in the present study, it is possible to identify two possible explanations and suggest fruitful avenues for future research. The first explanation is that the ECPs did not always provide a clear assessment of what would happen next for each of the patients they visited, and that a lack of clarity undermined the recovery of these patients and increased their risk of requiring or seeking hospital-based care. The second explanation is that health conditions which are more difficult to assess and explain to patients are less likely to improve and more likely to require additional, hospital-based care. Certainly, patients with lower self-reported health status have been found to be significantly less satisfied with their out-of-hours care in the past [16], and it is likely that it is difficult for ECPs *and *GPs to clearly assess or explain *all *conditions to *all *patients. Nonetheless, the present study found that ECPs' assessments can be unclear to out-of hours primary care patients, despite the fact that they spend longer with them than GPs. Further research is therefore warranted to establish whether this might reflect ECPs' relative inexperience in out-of-hours primary care, and whether this might improve with appropriate experience, clinical supervision or training. Further research is also required to establish if a lack of clarity is only found when ECPs provide such care, or whether it might be expected from any practitioner dealing with difficult conditions or patients with confusion. Either way, additional support and training might be appropriate for ECPs and GPs to improve patient understanding of the out-of-hours assessments they provide.

## Conclusion

The present study found high levels of agreement with the care provided or self-reported compliance with advice provided by ECPs during out-of-hours primary care home visits. However, it also found a sizeable minority of patients who were not clear about their ECP's assessment, and this was associated with subsequent wellbeing and use of services.

## Competing interests

None. There was no specific funding for the study. MH and TM carried out the study whilst employed at London Ambulance Service NHS Trust.

## Authors' contributions

MH, TM and DM designed the study. TM carried out the patient interviews. MH, TM and GTHE carried out the data analysis and interpretation. MH led preparation of the paper with GTHE. TM and DM commented substantially on all drafts.

## Pre-publication history

The pre-publication history for this paper can be accessed here:


